# Clinical Features of Congenital and Developmental Cataract in East China: A Five-year Retrospective Review

**DOI:** 10.1038/s41598-017-04332-1

**Published:** 2017-06-26

**Authors:** Xiangjia Zhu, Yu Du, Wenwen He, Ting Sun, Yinglei Zhang, Ruiqi Chang, Keke Zhang, Yi Lu

**Affiliations:** 1 Department of Ophthalmology, Eye and Ear, Nose, and Throat Hospital of Fudan University, 83 Fenyang Road, Shanghai, 200031 China; 2 Key Laboratory of Myopia, Ministry of Health, 83 Fenyang Road, Shanghai, 200031 China; 3 Eye Institute of Eye and Ear, Nose, and Throat Hospital of Fudan University, 83 Fenyang Road, Shanghai, 200031 China; 4 Key Laboratory of Visual Impairment and Restoration of Shanghai, Fudan University, 83 Fenyang Road, Shanghai, 200031 China; 5 Department of Biostatistics, School of Public Health, Key Laboratory of Public Health Safety, Ministry of Education, Fudan University, 130 Dong’an Road, 200032 Shanghai, China

## Abstract

Congenital/developmental cataract is a significant cause of blindness in children worldwide. Full knowledge of clinical features is essential for early diagnosis and proper treatment to prevent irreversible visual impairment. We conducted a retrospective chart review on 520 congenital/developmental cataract cases based on a five-year clinical data from Eye and ENT Hospital of Fudan University, Shanghai, China. Clinical features including age at the surgery, chief complaints, interval between initial identification of cataract-related manifestations and surgery, etc. were summarized. 56.3% of children were bilateral. The age at surgery ranged from 0.25 to 17.4 years, only 9.2% receiving surgery below 1 year. Interval between initial identification of manifestations and surgery ranged from 2 days to 17 years. Concomitant congenital abnormalities were present in 67 patients, with persistent hyperplastic primary vitreous and congenital heart disease as the most frequent ocular and systemic disorders. Strabismus and nystagmus were seen in 20.6% and 11.9% of patients. In bilateral cataract patients with strabismus, axial lengths of esotropia-affected eyes were statistically shorter than exotropia-affected eyes. These findings provide information on characteristics of congenital/developmental cataract in China and may assist in achievement of comprehensive treating strategies in these cases.

## Introduction

Congenital/developmental cataract is a main cause of childhood blindness, the prevalence of which was reported to be as high as 1.7 to 14.7 per 10,000 children in Asia area^1, 2^. Early diagnosis and timely treatment is crucial for preventing irreversible visual impairment among them. Therefore, it is very important to analyze the preoperative clinical characteristics of congenital/developmental cataract in detail for the proper planning of comprehensive strategies in dealing with childhood cataracts.

However, there are few studies about the clinical features of these patients undergoing surgery^3–5^, especially in Asian countries. As the best and the largest eye hospital that carries out pediatric cataract surgery in East China, we reported the clinical features of congenital/developmental cataracts based on a five-year single center experience of 520 cases in Eye and Ear Nose Throat (ENT) Hospital of Fudan University.

## Results

### Baseline information

Of all the 520 congenital/developmental cataract patients, 314 (60.4%) were boys and 293 (56.3%) were bilateral cataract. The age of the cataract patients at surgery ranged from 0.25 to 17.4 years, with the average of 4.74 ± 3.36 years. Fifty-nine (11.3%) cases were identified with family history of congenital cataract. More cases with family history were seen in bilateral cataracts than in unilateral cases (P < 0.001, Chi-square test). Chief complaints reported by parents or children themselves at admission were obvious leukocoria, significant decrease in visual acuity, or strabismus, which respectively accounted for 38.8%, 41.3%, and 18.1% of all the children. And 9 children came to our hospital with the complaint of occasional identification of cataract by the vision screening at school.

Tables 1 and 2 separately showed the distribution of age at surgery and the interval between the first identification of cataract-related manifestations and surgical treatment in congenital/developmental cataract patients reviewed in this study. As indicated, 9.2% (48/520) of patients received cataract surgery within the first year since birth and 56.2% (292/520) of patients aged one to five years old when having the surgery. Interval between initial identification of cataract-related manifestations and surgery ranged from 2 days to 17 years. Only 6.9% (36/520) received timely surgical treatment within one month since the identification of abnormal manifestations, 49.8% (259/520) waited for one to five years for the treatment and 20% (104/520) even waited for more than five years for the surgical treatment.

**Table 1:** Distribution of ages at surgery in congenital/developmental cataract patients.

Age	Cataracts operated
≤2 m	0 (0.0%)
>2m–1y	48 (9.2%)
>1y–2y	70 (13.5%)
>2y–3y	87 (16.7%)
>3y–4y	68 (13.1%)
>4y–5y	67 (12.9%)
>5y–10y	133 (25.6%)
>10y	47 (9.0%)

Values are presented as n and percentage of all the congenital/developmental patients reviewed in this study in the bracket.

m = month; y = year.

**Table 2:** Distribution of interval between initial identification of cataract-related manifestations and surgery in congenital/developmental cataract patients.

Time	Between onset of abnormal manifestion and surgery
≤1m	36 (6.9%)
>1m–1y	121 (23.3%)
>1y–2y	75 (14.4%)
>2y–3y	76 (14.6%)
>3y–4y	59 (11.3%)
>4y–5y	49 (9.4%)
>5y–10y	86 (16.5%)
>10y	18 (3.5%)

Values are presented as n and percentage of all the congenital/developmental patients reviewed in this study in the bracket.

m = month; y = years.

### Axial length

Axial lengths in the affected eyes of unilateral cataract children were statistically longer than those of the fellow eyes: 22.30 ± 1.87 mm versus 22.08 ± 1.29 mm (P = 0.032, Paired-sample t test). Axial length difference between the cataractous eyes and the fellow eyes was positively correlated with the interval between diagnosis and cataract surgery (Correlation coefficient: 0.153, P = 0.034, Spearman Correlation). While in cases of bilateral cataract, no statistically significant difference was seen between axial lengths of both eyes: 22.01 ± 2.35 mm versus 21.95 ± 2.23 mm (P = 0.193, Paired-sample t test).

### Concomitant abnormalities

Of all the congenital/developmental cataract children, 12.9% (67/520) had concomitant abnormalities (ocular: 44, systemic: 17, both:6). In 31 unilateral cataract patients with comorbidies, 24 had concomitant oculopathies, 5 had systemic disorders and 2 had both. In 36 bilateral cataract patients with comorbidies, 20 had concomitant oculopathies, 12 had systemic disorders and 4 had both. In bilateral cataract patients with concomitant abnormalities, the percentage of systemic disorders (16/36, 44.4%) was greater than that in unilateral cases (7/31, 22.6%, P = 0.06, Chi-square test).

Ocular and systemic abnormalities accompanying cataract were listed in Table 3. The most frequently seen ocular abnormalities were persistent hyperplastic primary vitreous (PHPV) and retinochoroidal coloboma. All the PHPV presented in the affected eyes of unilateral cataract, with statistically shorter axial length than non-cataractous eyes (19.35 mm versus 21.43 mm, P < 0.001, Paired-sample t test). The most frequently seen systemic disorders were congenital heart disease and cleft lip and palate.

**Table 3:** Congenital ocular and systemic abnormalities associated with congenital/developmental cataracts.

Ocular	Number of patients	Systemic	Number of patients
PHPV	17	Congenital heart disease	14
Retinochoroidal coloboma	13	Cleft lip and palate	3
Microphthalmos	4	Ventral hernia	1
Congenital glaucoma	2	Intussusception	1
Abnormalities of cornea:		Dysaudia	1
Microcornea	6	Developmental retardation	1
Corneal dermoid tumor	1	Epilepsy	1
Peters anomaly	2	Aplastic anemia	1
Abnormalities of iris:			
Aniridia	4		
Congenital hyperplastic pupillary membranes	4		
Heterochromia iridis	1		

Values are presented as n.

### Strabismus and nystagmus

Of all congenital/developmental cataract children reviewed, 20.6% (107/520) were diagnosed with strabismus. Similar occurrence of strabismus was found in unilateral and bilateral cataract patients: 23.7% (54/228) in unilateral cases and 18.2% (53/292) in bilateral cases (P = 0.12, Chi-square test). Proportion of esotropia: exotropia in unilateral cataract patients were 15:39 and 32:21 in bilateral cases. More exotropia was seen in unilateral cataractous eyes and more esotropia in bilateral cases (exotropia: 72.2% versus 39.6%, esotropia: 60.4% versus 27.8%; P = 0.001, Chi-square test). In bilateral cataract patients with strabismus, axial lengths of esotropia-affected eyes were statistically shorter than exotropia-affected eyes (eyes were randomly selected, 21.05 ± 2.07 mm verse 25.50 ± 3.98 mm, P < 0.001, Student’s t test).

Besides, of all the patients, 11.9% (62/520) had nystagmus. Bilateral cataract eyes were accompanied with statistically more nystagmus than unilateral cataract (P < 0.001, Chi-square test). Additionally, there were 5.2% (27/520) of patients who had both strabismus and nystagmus, 20 of which had bilateral cataract.

### Surgical techniques and visual outcomes

Of 520 children, 418 (80.4%) underwent cataract extraction and anterior vitrectomy with primary intraocular lens (IOL) implantation. 76.4% of implanted IOL were Acrysof SA60AT single-piece IOL (Alcon, Texas, USA) and 23.6% were Tecnis ZCB00 one-piece IOL (Abbott Medical Optics, California, USA). Seven children with posterior polar cataract were reported to have an inevitable posterior capsular rupture during cataract extraction, which were successfully managed by anterior vitrectomy. And iris of two children were accidentally injured during surgery due to the microcoria, yet were also well managed intraoperatively. Totally only 265 (50.9%) children cooperated in both preoperative and postoperative visual acuity tests, aged 7.86 ± 4.11 years averagely. And in this proportion of children, visual acuity was averagely 1.02 ± 0.54 LogMAR before the surgery and improved to 0.64 ± 0.43LogMAR at the first postoperative day.

## Discussion

Understanding the preoperative clinical features of congenital/developmental cataract is very useful for the planning of comprehensive treatment strategies of this vision threatening eye disease. However, studies in large sample size are rare in Asian countries, especially in China. The Eye and ENT Hospital of Fudan University is the largest tertiary referral center for children in East China, which receives almost all the pediatric cataracts in this region. Based on the five-year clinical data of pediatric cataracts in our center, we summarized the common clinical presentations of congenital/developmental cataract in east China.

Timely clinical interventions for pediatric cataracts are of prime importance for good visual outcomes. Significant correlation between the axial length difference of affected and fellow eyes and the time since the initial identification of cataract-related manifestations in unilateral cataract children was found in our study. Although weak with the coefficient of 0.153, it still implied that timely treatment was important to reduce the imbalance between the ocular developments of two eyes, which may require further verification in larger sample size. Besides, as Hartmann *et al*. reported, younger age at cataract surgery contributed to better visual outcomes in congenital cataracts^6^. However, in our study population, no children received cataract surgery within two months since birth, similar to the findings as reported by You *et al*. in ref. 7. Perhaps due to the different onset ages of developmental cataract, the distribution of age at surgery in our study was quite scattered.

As for the interval between the initial identification of cataract-related manifestations and surgery, only 30.2% (≤1 m: 6.9%; >1 m–1y: 23.3%) of the patients had surgery within one year, while others experienced longer delay, 20% even more than five years. According to the chief complaints shown in medical records, 98.2% (38.8% + 41.3% + 18.1%) of children had suffered from obvious leukocoria, significant decrease in visual acuity, etc. for varying durations ranging from 2 days to 17 years. Hence, it could be seen the vast majority of congenital/ developmental cataract children in East China experienced delay in proper treatment. This phenomenon might be in part due to the lack of sound vision screening system for children in China and might be also attributed to the regional or family economic woes, since the accessibility to cataract surgery was closely related to economy, as indicated by multiple epidemiological investigations worldwide^8, 9^. Moreover, low level of economic circumstances is very likely to be consistent with low level of health education, resulting in parents’ unawareness of children’s abnormal manifestations, which may also lead to the prevalent delay of the illness. Given that the east area is the most developed part of China, patients in other less developed regions were very likely to experience much longer delay in treatment and consequently more undesirable prognosis.

In the study population, we found 12.9% with congenital comorbidities, oculopathies accounting for 74.6% of them. Seventeen patients were identified with monocular PHPV with the shorter axial length in the affected eye and the corresponding prevalence was 3.3%, occurring the most times among all the concomitant congenital oculopathies, which was consistent with results from previous studies, such as 2.0% reported by Toshiyuki *et al*. in Japan^10^, 3.8% reported by Yang *et al*. in Taiwan^5^ and 3.0% reported by Plager *et al*. in the USA^11^. The second most common concomitant oculopathy was retinochoroidal coloboma, a rare ocular malformation resulting from defective closure of the embryonic fissure in our study, with a prevalence of 2.5%, suggestive of abnormal embryogenesis associated with congenital cataracts^12^. Besides, microcornea or microphthalmos were found in 1.9% of our cases (2.3% of cataractous eyes), similar to the 1.67% reported by Lin *et al*. in Guangdong Province, the southern part of China^13^ and lower than previous reports of 8.6% by Toshiyuki *et al*.^10^ With regard to associated systemic disorders, congenital heart disease was the most frequent systemic abnormality; cleft lip and palate came second in our study. Toshiyuki *et al*.^10^ found that prevalence of associated systemic problems was significantly higher among children without family history of cataract than those with, which was not identified in our study.

Another aspect was that larger proportions of patients were identified with ocular disorders such as strabismus and nystagmus. The prevalence of strabismus in our study was 20.6%, which was similar to 19% reported by Fakhoury *et al*. in France^14^, but was lower than the report of 28% by Kim *et al*. in Korea^15^ and higher than the report of 9.5% by Lin *et al*. in Guangdong Province of China^13^. Dermirkilinc *et al*. reported that unilateral cataract cases in children were more prone to develop strabismus^16^, which was not identified in our study. More exotropia was seen in the cataractous eyes of unilateral cataract patients, while in patients with bilateral cataract, esotropia was more prevalent. In those with unilateral cataract along with exotropia, a possible explanation was that the cut off from essential light due to cloudy lens in the early age retarded the normal development of retinal function and corresponding striate cortex circuitry in infants, which left the cataractous eye exotropic, as Frank *et al*.^17^ reported infants did not start to show convergence until six weeks of age. One interesting finding about axial lengths of strabismic eyes was that among bilateral cataract patients with strabismus in our study, axial lengths of esotropia-affected eyes were shorter than exotropia-affected eyes. This agreed with finding of Kim *et al*.^18^ that among patients with sensory horizontal strabismus, axial length was somewhat longer in exotropia group. And similarly, Tanaka *et al*.^19^ reported in their study that among patients with extremely long axial length concomitant with strabismus, exotropia was much more frequently seen. Additionally, nystagmus was found in as many as 11.9% of patients. More nystagmus was seen in bilateral cataract due to severer vision impairment compared to unilateral cataract. Both strabismus and nystagmus were signs of severe vision deprivation early in life^20^, indicating unfavorable visual outcomes of cataract patients with these disorders. Particularly for the 5.2% patients with both strabismus and nystagmus in our study, prognosis might be even worse.

In conclusion, even in the developed east China, patients diagnosed with congenital/developmental cataract were very likely to experience delays in treatment. Concomitant abnormalities are frequently seen in congenital/developmental cataract patients, which should be taken into consideration for making comprehensive treating strategies.

## Patients and Methods

The Institutional Review Board of the Eye and ENT Hospital of Fudan University, Shanghai, China approved this retrospective chart review, which was affiliated to Shanghai Pediatric Cataract Study (registered at www.clinicaltrials.gov, accession number NCT03063216). All procedures were conducted in agreement with tenets of the Declaration of Helsinki. Written consent forms were acquired from guardians of the patients for the use of their medical data for research purpose.

### Patients and clinical records reviewed

We reviewed medical records of congenital/developmental cataract patients who underwent cataract extraction with or without IOL implantation between April 2009 and April 2014 in our hospital. Those with complete medical records were eligible for inclusion.

Basic information of the patients including gender, family history, laterality, chief complaints, the age at surgery and the interval between the initial identification of cataract-related manifestations and surgery were reviewed.

Preoperative examinations of congenital and developmental cataract children included visual acuity test, slit-lamp examination through dilated pupil for the confirmation of diagnosis, axial length measurement by IOLMaster or intraoperative applanation A-scan ultrasonography for the calculation of IOL power, and B-scan ultrasonography for the inspection of posterior segment abnormalities. Preoperative visual acuity, axial length and concomitant congenital abnormalities including oculopathies such as strabismus and systemic disorders were analyzed.

All the surgeries were performed under general anesthesia. For children who underwent cataract extraction by irrigation/aspiration device, surgical procedures were mainly making scleral tunnel incision, continuous curvilinear capsulorhexis, and cataract removal. A foldable IOL was primarily implanted into the capsular bag in selected cases. And no matter with IOL implanted or not, posterior capsulotomy and anterior vitrectomy were finally performed using a 23 G/25 G vitrectomy instrument. For children who underwent cataract extraction by 23 G/25 G vitrectomy instrument, surgical procedures were mainly making limbal incisions by a 23/25 G trocar with a microcannula, central capsulotomy, and cataract removal. And foldable IOL was primarily implanted into the capsular bag in selected cases. No matter with IOL implanted or not, posterior capsulotomy and anterior vitrectomy were then performed using the vitrectomy instrument. Surgical techniques used and the percentage of IOL implantation in these children was summarized as well as surgical complications. Besides, visual acuity on the first postoperative day were routinely documented and also analyzed in our study. Visual acuity results were converted to the logarithm of the minimum angle of resolution (LogMAR) for analysis.

### Statistical Analysis

Student’s t test or Paired-sample t test were utilized to compare the continuous variables and Chi-square test was used to compare categorical variables where appropriate. Spearman correlation analysis was used to analyze the association between axial length differences of the two eyes in unilateral cataract children and the interval between diagnosis and treatment. Values were presented as the mean ± standard deviation. P-value < 0.05 was considered to be statistically significant. Statistical analysis was carried out using IBM^®^ SPSS^®^ Statistics version 22 (IBM Corp., Armonk, New York, USA).
